# The Legislature vs. the Physicians

**Published:** 1891-08

**Authors:** 


### Editorial.

### THE LEGISLATURE *vs.* THE PHYSICIAN.

[The Lower House of Georgia's rural Legislature, in present session assembled, recently passed the following bill :

SECTION 1. *Be it enacted by the General Assembly of Georgia*, That from and after passage of this Act, no physician or prescription clerk in any drug establishment in the State shall be allowed to practice medicine or fill a prescription who may become drunk from use of either intoxicating liquor or opiates.

SEC. 2. *And be it further enacted*, That violation of the first section of this Act shall be a misdemeanor, and upon conviction shall be fined not less than two hundred dollars, nor more than five hundred dollars; and shall be liable for all damages to their patients or customers while practicing their profession or calling, while under the influence of intoxicants; and upon a second conviction of drunkenness shall forever forfeit their licenses, or the privilege under the laws of Georgia to practice medicine or fill prescriptions.

As we go to press this measure awaits the consideration of the Senate. The following communication has been received from a prominent physician in Atlanta. We commend it to our professional readers, and also to the thoughtful and prayerful contemplation of our rustic lawmakers.—L. B. G.]

You ask me what I think of this class legislation recently enacted by the Legislature of Georgia. It is iniquitous. Yes, it is iniquitous, officially to brand us physicians as a class especially addicted to the evil of intemperance to the exclusion of all other classes !

Every calling has its duties and responsibilities. Every man has a part to perform in this world, and whoever underrates his avocation forfeits his manhood. If a physician has great responsibilities involving life, property, etc., so has the railroad man, the navigator, the civil and mining engineer, the street-car driver, the brick-layer, the electrician, the lawyer, the divine, the judge, all trades and professions, and last but not least, the *legislator !*

It would be interesting to compare the moral status of physicians with that of any other class of citizens. I think we would soon realize that comparisons are odious!

Our dealings are essentially with suffering humanity. Who does more charity than the physician? Who does more for the honor, good name and protection of home and family than the physician? Were we to betray the confidences that we are entrusted with, this world would be a field of carnage! Who is oftener treated with ingratitude than the physician?

To deprive a physician of his inalienable right to make a living because, forsooth, he has had the misfortune to get intoxicated, either by himself or with such a companion as a lawyer, a capitalist, a tramp, or perhaps a legislator, is a monstrosity!

A minister *may* have the misfortune of getting drunk. I think it will be conceded that this is not an impossibility. The church may or may not take cognizance of his offence. He may be deposed or he may be shielded by his flock. Is there a law to punish him or withdraw his license from him?

A lawyer may go to a club dining, or to a noted banquet—he always manages to get an invitation—and he will go home full to the brim. Is there a law debarring him from the right of practicing law?

A judge, the very incarnation of the majesty of the law, may, after having during a limited session sent a poor inebriate to the chaingang over and over again for the same offence and deprived his wife and children of his support, go and rest his worried brow in the shades of Tybee, Cumberland or St. Simon's, and there and then make up for lost time and opportunity, and silently but surely and unmistakably imbibe *usque ad nauseam*, whilst his friends are fishing or bathing on the seashore. Is there any law to render his conduct odious in the eyes of his fellow-men and disgrace him by removing him from office ?

And what shall I say of our legislators themselves, entrusted with the interests of this great commonwealth ? Are they, as a class, above reproach ? If any one wishes to be enlightened and edified on this subject, let him visit the numerous bar-rooms of this city, and I will venture the assertion that for one doctor found or seen in front of the counter, he will find ten legislators. There are no less than two hundred physicians in Atlanta.

I will close by stating that the legislators have been for several years encircling their noble heads with a halo of glory. Their persons are inviolable, and the police are not permitted to arrest any of them. "*The necessity for a quorum demands it.*"

"*Ne touchez pas a la reine*"—Do not touch the queen, says the French. Do not arrest a drunken law-maker, says the Georgia enactment ; but *if a doctor gets drunk let him and his family starve !*

"It is not for kings, O Lemuel, it is not for kings to drink wine, nor for princes, strong drink. Lest they drink, and forget the law, and pervert the judgment of any of the afflicted."

